# Supporting Clinical Competencies in Men’s Mental Health Using the Men in Mind Practitioner Training Program: User Experience Study

**DOI:** 10.2196/48804

**Published:** 2023-11-07

**Authors:** Zac E Seidler, Ruben Benakovic, Michael J Wilson, Justine Fletcher, John L Oliffe, Jesse Owen, Simon M Rice

**Affiliations:** 1 Orygen Melbourne Australia; 2 Centre for Youth Mental Health The University of Melbourne Melbourne Australia; 3 Movember East Melbourne Australia; 4 Centre for Mental Health School of Population and Global Health The University of Melbourne Melbourne Australia; 5 School of Nursing University of British Columbia Vancouver, BC Canada; 6 Department of Nursing The University of Melbourne Melbourne Australia; 7 Department of Counselling Psychology University of Denver Denver, CO United States

**Keywords:** e-learning, mental health services, psychotherapy, men’s mental health, masculinity

## Abstract

**Background:**

Engaging men in psychotherapy is essential in male suicide prevention efforts, yet to date, efforts to upskill mental health practitioners in delivering gender-sensitized therapy for men have been lacking. To address this, we developed Men in Mind, an e-learning training program designed to upskill mental health practitioners in engaging men in therapy.

**Objective:**

This study involves an in-depth analysis of the user experience of the Men in Mind intervention, assessed as part of a randomized controlled trial of the efficacy of the intervention.

**Methods:**

Following completion of the intervention, participants provided qualitative (n=392) and quantitative (n=395) user experience feedback, focused on successes and suggested improvements to the intervention and improvements to their confidence in delivering therapy with specific subpopulations of male clients. We also assessed practitioner learning goals (n=242) and explored the extent to which participants had achieved these goals at follow-up.

**Results:**

Participants valued the inclusion of video demonstrations of skills in action alongside the range of evidence-based content dedicated to improving their insight into the engagement of men in therapy. Suggested improvements most commonly reflected the desire for more or more diverse content, alongside the necessary adaptations to improve the learning and user experience. Participants also commonly reported improved confidence in assisting men with difficulty articulating their emotions in therapy and suicidal men.

**Conclusions:**

The evidence obtained from this study aids in plans to scale Men in Mind and informs the future development of practitioner training interventions in men’s mental health.

**International Registered Report Identifier (IRRID):**

RR2-10.1186/s40359-022-00875-9

## Introduction

### Men’s Mental Health and Help-Seeking Experiences

The intersection between men’s mental health outcomes, particularly suicide, and masculine gender socialization has drawn increasing research attention [1]. In many countries, traditional gender norms that dictate that men should be emotionally stoic and self-reliant are thought to manifest as barriers to men’s help-seeking for mental health care [2]. Such barriers are thought to subsequently amplify men’s vulnerability to suicide [3]. To date, much of the empirical literature has focused on tackling the issue of improving men’s access to targeted and tailored evidence-based interventions [4,5]. In doing so, one of the keys to improving care is tailoring practitioner training within a larger landscape of culturally adapted treatments [6,7].

Prior research highlights that men seeking help for depression often receive insufficiently engaging services that can exacerbate shame or feelings of alienation in the therapy environment, leading to high rates of premature dropout [8,9]. This mismatch is also reflected in suicidal men who seek help, with treatment often neglecting men’s agency and autonomy, thereby not meeting their needs and directly affecting their efforts and desire to seek further support [10,11]. Research exploring the experiences of mental health practitioners substantiates common challenges when working with men, including, for example, a lack of preparedness to tap into traditionally masculine men’s emotional worlds [12]. Importantly, interventions aiming to lever men’s rigid adherence to traditional masculine norms to promote help-seeking have been shown to increase help-seeking intentions [13]. Public health promotion campaigns aimed at affirming men’s help-seeking behavior have also demonstrated positive effects by increasing help-seeking intentions (eg, Man Up [14]), awareness, and behavior (eg, Man Therapy [15] and Real Men. Real Depression [16]). However, with this intended increase in male help seekers, comparable efforts are needed to ensure that practitioners working with men in therapy are equipped to sensitize their treatment to better reach, respond, and retain men in all their diversities. Indeed, this is even more critical in the case of suicidal men, where practitioners may only have a short window of opportunity to effectively engage their male clients [17].

### Practitioner Education for Engaging Men in Therapy

Currently, guidelines for engaging men in therapy do exist [18], alongside case studies documenting the sensitization of therapy for men [19]. The existing literature has so far provided consistent recommendations for working with men in practice, advocating for practitioners to have an awareness and understanding of how gender socialization impacts male client presentation and the therapeutic relationship, to self-reflect on their own gender biases and socialization, and to implement specific microskills around communication and treatment structures that adapt their practice to be more male oriented [6]. However, the field currently lacks a synthesis of these findings into accessible and scalable training initiatives for the mental health workforce [6].

To address this gap, Seidler et al [20] created Men in Mind, a web-based training program designed to upskill mental health practitioners (eg, psychologists, counselors, and social workers) to engage and respond effectively to men in psychotherapy. Men in Mind was initially evaluated through a pilot trial among 196 Australian practitioners, with the results providing evidence for the acceptability, feasibility, and potential efficacy of the program [21]. Subsequently, a randomized controlled trial (RCT) [22] was conducted among 587 practitioners with results showing that Men in Mind was effective at increasing practitioners’ self-reported efficacy to engage and respond to men in psychotherapy to a large effect (Cohen *d*=2.63, 95% CI 2.39-2.87; *P*<.001).

### Men in Mind: Next Steps

Importantly, efficacy alone does not ensure real-world applicability or feasibility, which must be a core consideration when designing and evaluating interventions for subsequent scaling and dissemination. Indeed, meta-analytic evidence suggests that learning experiences in web-based environments can moderate the differences between learning outcomes when comparing web-based and face-to-face learning [23]. These results also suggest that web-based learning may increase student performance when compared with face-to-face instruction. This is particularly significant given the potential for e-learning training programs (web-based learning initiatives [24]) to be efficient and easily scalable. However, it is rare for e-learning evaluation studies to conduct dedicated in-depth analyses of learners’ experiences beyond simple user experience feedback [25]. This is a notable gap when considered alongside mixed evidence regarding knowledge retention in web-based learning formats: improving web-based learning experiences could be critical to practitioner knowledge retention. Specifically, Burn et al [26] found that web-based practitioner training for engaging fathers in parenting interventions led to decreases in practitioner competencies at follow-up (where the same decreases were not observed in in-person training). If Men in Mind is to be scaled across international markets to assist practitioners, it is essential to understand participants’ experiences of the successes and shortcomings of the intervention via dedicated analysis. This will complement prior quantitative evidence of the efficacy of Men in Mind to provide a detailed understanding of how and why the intervention improved practitioner self-efficacy, thereby informing broader international efforts to achieve knowledge translation in the development of gender-tailored mental health interventions for men [27]. The expectations and goals of the practitioners undertaking the training are also of value here. Clarifying whether the learnings of Men in Mind are in line with practitioner expectations and whether they will help them achieve their goals is critical in ensuring motivation and engagement with the program. To address these gaps in user experience, this study aims to provide an in-depth report of participant experience during the Men in Mind trial, reporting our qualitative evaluation of participants’ experiences of the intervention, quantitative e-learning feedback, and learning implementation goals.

## Methods

### Study Design

This study involved the analysis of training feedback and goal assessment outcomes of the Men in Mind RCT, a web-based RCT with 2 parallel groups, a Men in Mind group (who underwent the Men in Mind training program) and a waitlist control group (who underwent the training program after the Men in Mind group). The RCT examined the efficacy of Men in Mind in improving practitioners’ clinical competencies related to engaging with and responding to male clients in therapy. The protocol for this trial has been described in detail elsewhere [28], with the primary and secondary quantitative outcomes forthcoming. All relevant documentation regarding the trial was preregistered and available at the Australian New Zealand Clinical Trials Registry (ACTRN12621001669886).

### Ethical Considerations

Ethics approval was obtained from the University of Melbourne Psychology, Health, and Applied Sciences Human Ethics subcommittee (22618). All participants provided informed consent at the beginning of the web-based survey before participating. All data provided was deidentified. No financial compensation was provided to participants for taking part in this study; their free access to the Men in Mind training intervention was considered adequate compensation for their time.

### Participants and Procedure

Participants were Australian-based mental health practitioners who were recruited into the Men in Mind RCT. All the participants were fluent in English and were currently delivering psychotherapy to male clients. A “mental health practitioner” was defined as a client-facing mental health professional currently delivering psychotherapy (eg, psychologists, psychiatrists, counselors, or social workers). Participants were recruited from a pool of interested practitioners who registered an interest in the study via a web-based portal following advertisements (delivered via Facebook and the website of Orygen, the study sponsor) and presentations by the research team. Participants were excluded if they were an undergraduate student at the time of the trial. Informed consent was provided by the participants via the web before the first data collection point and was required for participation.

There were 3 data collection points in the trial: baseline, primary end point (6 weeks following randomization), and follow-up (12 weeks after the primary end point for the Men in Mind group and 6 weeks after completion of the Men in Mind group by those in the waitlist control group). Follow-up occurred at different time points for both groups as it was designed as a follow-up assessment for the Men in Mind group and a comparative postintervention assessment for the waitlist control group (to be compared with the primary end point for the Men in Mind group). This paper focuses on feedback and goal assessment data regarding the Men in Mind intervention from both groups, across the primary end point and follow-up data collection points.

### Intervention

Regarding the intervention itself, Men in Mind is a self-led web-based training program for mental health practitioners, which aims to upskill their self-reported clinical competencies related to engaging and responding to male clients in therapy. The content of Men in Mind comprises five modules: (1) “Rebranding Masculinity”—which offers in-depth understandings of men’s gender socialization, masculinities, and connections between masculine norms and men’s mental health; (2) “Your Gender, Your Practice, Your Rules”—lobbying practitioners to reflect on their gender socialization and how this might impact on their engagement of male clients; (3) “The Hook: Engagement and Motivation”—details strategies for engaging and motivating male clients, alongside tools for assisting men experiencing difficulty identifying or articulating their emotions; (4) “The Depressed Man”—aims to equip practitioners with the tools to identify externalizing profiles of male depression, particularly responding to anger and irritability; and finally, (5) “The Suicidal Man: Saving Those Thousand Lives”—shares connections between masculine socialization and men’s suicide, highlighting warning signs and tools for therapeutically engaging suicidal men. These modules, along with all content within Men in Mind, are all evidence based and have been iteratively developed through past research, which has included a Delphi expert consensus study [29], qualitative research exploring male clients and practitioners’ perspectives [9,30], and a scoping review regarding engaging men in psychological treatment [31].

### Outcomes

#### Overview

Sociodemographic characteristics of the participants were collected at baseline. Table 1 specifies the outcome assessment procedures across time points of the trial.

**Table 1:** Timing of outcomes assessed across trial end points.

		Men in Mind group		Waitlist control group
		T2 (post)^a^	T3 (follow-up)	T3 (post)^a^
Five-item quantitative feedback		✓	✓	✓
Three-item qualitative feedback		✓		✓
**Goal assessment outcomes**				
	Learning goals	✓		
	Goal achievement		✓	

^a^The Men in Mind group had their postintervention assessment at T2 (6 weeks after baseline), while the waitlist control group started the program immediately after T2 and had their postintervention assessment at T3 (12 weeks after baseline). For the Men in Mind group only, T3 (follow-up) occurred 18 weeks after baseline.

#### Quantitative Feedback Items

To assess their experience of the Men in Mind training, participants completed 5 training program feedback experience items adapted from previous e-learning evaluation studies [32]. On a scale from 1 (strongly disagree) to 7 (strongly agree), participants were asked the following items: “I believe my current clinical practice will improve as a result of completing Men in Mind”; “I would recommend Men in Mind to other mental health professionals/colleagues”; “After completing Men in Mind, I feel more equipped to work with male clients in therapy”; “After completing Men in Mind, I am looking forward to working with more male clients”; and “After completing Men in Mind, I have been better able to retain male clients who have agreed to a course of therapy.” Participants completed these items after the intervention. Participants in the Men in Mind group also completed a repeat of 2 of the feedback items (“My current clinical practice has improved as a result of Men in Mind,” and “After completing Men in Mind, I have been better able to retain male clients who have agreed to a course of therapy”) at follow-up to assess for any changes in these items over the 12 weeks after the intervention.

#### Qualitative Feedback Items

After the intervention, the participants were asked to respond to 3 free-text entry items to gauge their experience of the training program. This survey occurred following completion of the intervention for both groups (ie, 6 weeks following randomization for the Men in Mind group and 12 weeks following randomization for the waitlist control group).

Participants were asked, “In your own words, what was the best thing about the training program for you?” “In your own words, what do you think could be improved about the training program?” “In your own words, which population(s) of male clients do you feel more confident working with now, as a result of completing Men in Mind?” (eg, men experiencing suicidality, men experiencing difficulty with emotional communication, and sexual minority men).

#### Goal Assessment Outcomes

At the primary end point, participants in the Men in Mind group were asked to respond to the following open-text item: “Now that you’ve completed Men in Mind, what are your three main goals for implementing the training into your practice?” These goals were then presented back to the participants at follow-up. They were then asked to indicate whether they met their goals by responding with “No progress yet”; “Making progress”; or “Achieved” (coded as 1, 2, or 3, respectively).

### Data Analysis

Bivariate analyses (a chi-squared test for categorical variables and an independent samples 2-tailed *t* test for continuous variables) were conducted to examine any differences between participants who responded to the relevant items and those that did not for the quantitative, qualitative, and goal assessment data. Responses to the quantitative items were analyzed using SPSS Statistics (version 27; IBM), with descriptive statistics presented for each of the 5 quantitative items.

Responses to the 3 open-ended qualitative items were analyzed using inductive thematic analysis. This involved a 6-stage progress of coding and theme development in accordance with the guidelines by Clarke et al [33]. The responses were first read in detail to gain familiarity with the data, with all responses being downloaded into a spreadsheet for analysis. Initial codes were then identified using open coding by 2 authors (MJW and RB), and codes were developed to encompass similar responses. Cross-comparison of 10.2% (40/392) of the responses for each of the 3 items was undertaken by 2 authors (MJW and RB), with any disagreements being discussed and resolved. These initial codes were then sorted and merged into broad themes to form preliminary findings. Subsequent themes were then reviewed by the lead author (ZES) and were appropriately named and condensed. Throughout this process, selected themes (particularly those produced with low-frequency codes) were subsumed under higher-order themes to better represent the underlying thematic content. Finally, consensus on the themes and illustrative quotes was reviewed by all authors through meetings and collaborative writing of this paper.

For the goal assessment data, qualitative content analysis was used to form the overall goal categories [34]. This analysis involved the analytic stages of preparation (in-depth immersion in the data), organizing (initial coding and grouping of similar responses; collation of overlapping codes), and reporting (development of a conceptual map to represent the data). One author (MJW) conducted the content analyses of the goals data. The proportion of progress made by the participants was reported in simple frequencies.

## Results

### Sample Characteristics

A total of 587 participants were included in the original Men in Mind RCT (300 assigned to the Men in Mind group and 287 assigned to the waitlist control group) with demographic characteristics comparable across both groups (Table 2). Once the intervention had been completed, 395 participants completed the 5-item quantitative training program feedback (210 participants from the Men in Mind group and 185 participants from the waitlist control group), with a further 190 participants (Men in Mind group only) completing the additional 2 items at the follow-up assessment point (12 weeks after the intervention). Comparatively, 392 participants completed the qualitative feedback and 204 participants in the Men in Mind group completed the goal assessment data (waitlist control group did not undergo goal assessment). Bivariate analyses were conducted to examine any potential demographic differences between participants who completed the quantitative, qualitative, and goal assessment data and the original 587 participants. These analyses found no differences in variables of gender, profession, employment load, qualifications, workplace, or region (Multimedia Appendix 1). A significant difference was found in the years of experience (as a practitioner) variable for both the quantitative data (χ^2^_3_=8.02, N=587; *P*=.046), and qualitative data (χ^2^_3_=9.40, N=587, *P*=.02), with those who completed the feedback being more experienced. An independent samples 2-tailed *t* test revealed a significant difference in the age variable for completers compared with the noncompleters, with the completion group being older, for the quantitative completers (t_585_=3.38; *P*=.001), the qualitative completers (t_585_=3.31; *P*=.001), and the goal assessment completers (t_585_=2.25; *P*=.02).

**Table 2:** Baseline characteristics (N=587).

Characteristics			Waitlist control (n=287)		Men in Mind group (n=300)		All participants (N=587)
Age (years), mean (SD)			42.09 (12.3)		43.34 (12.7)		42.73 (12.5)
**Gender, n (%)**							
	Man	65 (22.6)		82 (27.3)		147 (25)	
	Woman	219 (76.3)		217 (72.2)		436 (74.3)	
	Self-identified gender	3 (1)		1 (0.3)		4 (0.7)	
**Experience (years), n (%)**							
	0-2	82 (28.6)		96 (32)		178 (30.3)	
	3-5	87 (30.3)		93 (31)		118 (20.1)	
	6-10	57 (19.9)		61 (20.3)		111 (18.9)	
	≥11	61 (21.3)		50 (16.7)		180 (30.7)	
**Employment basis, n (%)**							
	Full time	153 (53.3)		150 (50)		303 (51.6)	
	Part time	89 (31)		109 (36.3)		198 (33.7)	
	Casual or contractor	26 (9.1)		20 (6.7)		46 (7.8)	
	Other	19 (6.6)		21 (7)		40 (6.8)	
**Highest education level completed, n (%)**							
	Certificate 3^a^	0 (0)		1 (0.3)		1 (0.2)	
	Certificate 4^b^	9 (3.1)		8 (2.7)		17 (2.9)	
	Undergraduate degree	37 (12.9)		36 (12)		73 (12.4)	
	Undergraduate degree (Hons)	71 (24.7)		80 (26.7)		151 (25.7)	
	Master’s degree	145 (50.5)		157 (52.3)		302 (51.4)	
	Doctoral degree or PhD	25 (8.7)		18 (6)		43 (7.3)	
**Profession, n (%)**							
	Provisional psychologist	42 (14.6)		55 (18)		97 (16.5)	
	General psychologist	85 (29.6)		75 (25)		160 (27.3)	
	Clinical psychologist	47 (16.4)		46 (15.3)		93 (15.8)	
	Counselor or psychotherapist	77 (26.8)		76 (25.3)		153 (26.1)	
	Occupational therapist	3 (1)		4 (1.3)		7 (1.2)	
	Social worker	25 (8.7)		40 (13.2)		65 (11.1)	
	Nurse practitioner	6 (2.1)		4 (1.3)		10 (1.7)	
	Family therapist or practitioner	2 (0.7)		0 (0)		2 (0.3)	
**Clinical setting of practice, n (%)**							
	Public or community health service	49 (17.1)		37 (12.3)		86 (14.7)	
	Private practice	120 (41.8)		126 (42)		246 (41.9)	
	Hospital	7 (2.4)		12 (4)		19 (3.2)	
	Corporate organization	8 (2.8)		5 (1.7)		13 (2.2)	
	School, university, or other education service (eg, TAFE^c^)	37 (12.9)		37 (16.3)		74 (14.7)	
	Not-for-profit organization	37 (12.9)		49 (16.3)		86 (14.7)	
	Prison or correctional facility	12 (4.2)		15 (5)		24 (4.6)	
	Veterans’ mental health service	5 (1.7)		4 (1.3)		9 (1.5)	
	Government or government organization	12 (4.2)		15 (5)		27 (4.6)	
**Locale of clinical practice, n (%)**							
	Metropolitan	199 (69.3)		198 (66)		397 (67.6)	
	Regional	67 (23.3)		79 (26.3)		146 (24.9)	
	Rural or remote	21 (7.3)		23 (7.7)		44 (7.5)	

^a^Accredited minimum qualification course for entry, typically 1 to 2 years.

^b^Accredited course that prepares students for work in areas that may require complex skills, typically 6 months to 2 years.

^c^TAFE: technical and further education.

### Quantitative Feedback

**Figure 1:**
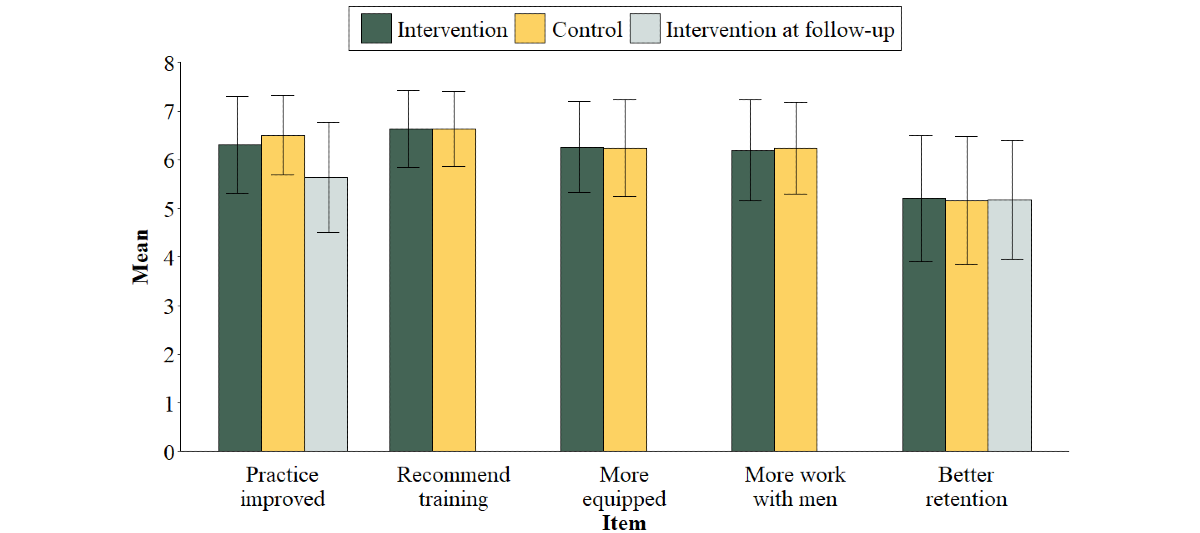
Five-item quantitative feedback of practitioners in the Men in Mind group and the waitlist control groups after the intervention and the Men in Mind group at follow-up.

Overall, the participants demonstrated a high level of agreement on the positive impact of Men in Mind (Figure 1) across the following items (combined group means): (1) improved clinical practice because of Men in Mind (mean 6.40, SD 0.92), (2) their likelihood of recommending the program (mean 6.63, SD 0.78), (3) feeling more equipped to work with male clients (mean 6.25, SD 0.96), and (4) increased desire to work with more men in therapy (mean 6.21, SD 0.99). These 4 posttraining feedback items (maximum score of 7) showed notable stability between the items and between the groups. For the last item, (5) being better able to retain male clients, the scores were lower (mean 5.18, SD 1.30), although they still demonstrated a moderate level of agreement and remained stable at the 12-week follow-up. There was a slight drop in the mean score for the improvement item at follow-up (mean 5.64, SD 1.13). There were no significant differences between the intervention and waitlist control groups, except for item 1 after the intervention (t_393_=2.04, *P*=.04).

### Qualitative Feedback

#### Best Elements of Men in Mind

**Figure 2:**
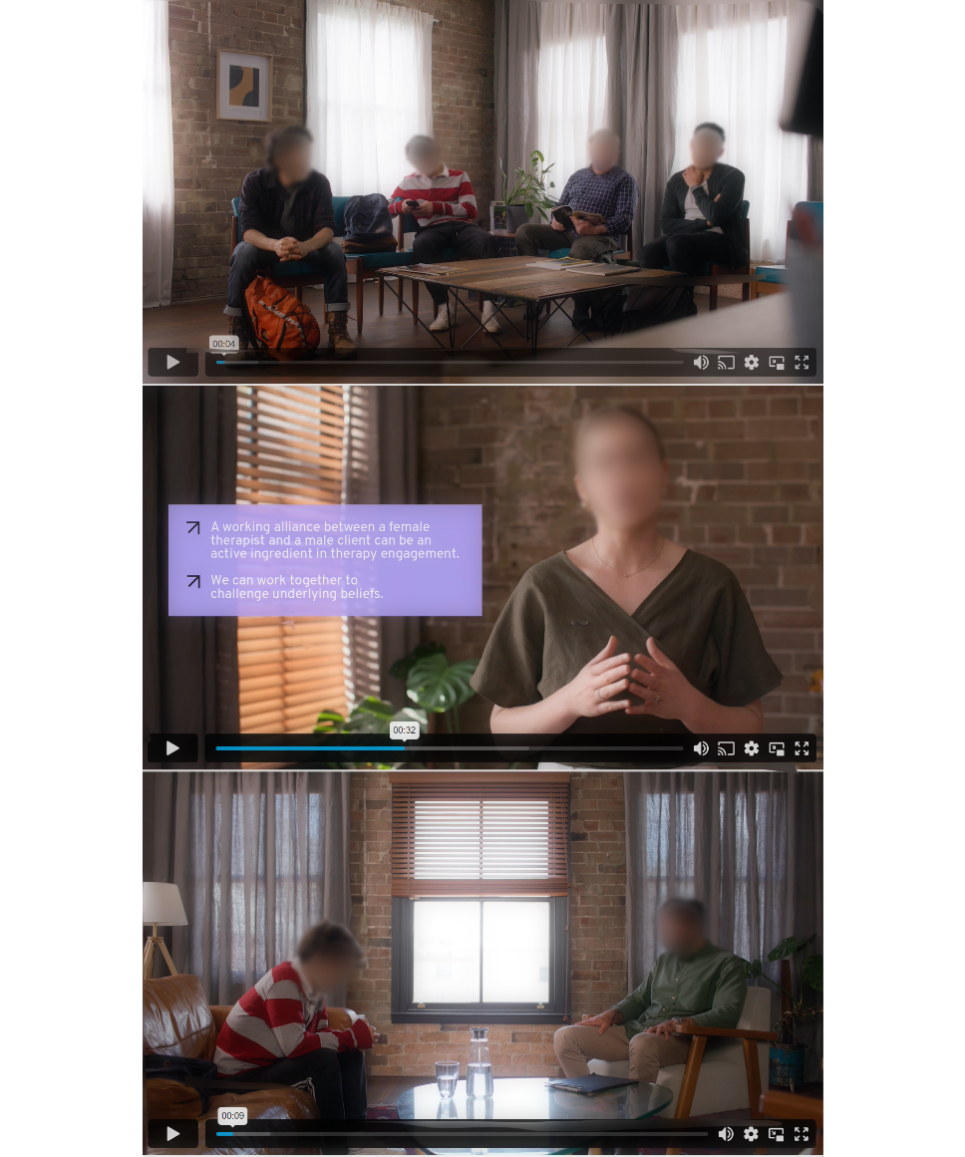
Depictions of the video content included throughout Men in Mind.

The most highly favored element of Men in Mind, among the 392 respondents, concerned the 40 video depictions (Figure 2) of the 4 male clients’ presentation and progress in therapy, alongside demonstration of “skills in action” to complement written content (Table 3). Participants’ appreciated the practical “examples of how to vocalise some of these important questions and conversations with clients in new ways,” coupled with informative comparisons between a “typical and then alternative strategy for working with the client” and the diversity of presenting issues and challenges depicted using the case studies: “seeing the four different role plays and the outcomes from them has given me the tools and information to work with client’s that present with any issue.”

**Table 3:** The thematic analysis map of participants’ responses to “What is the best thing about the training program for you.”

Group			Responses, n (%)^a^
**Facilitated demonstrations and video content**			
	Role-play videos of skills in action	146 (37.2)	
	Diverse client video examples	38 (9.7)	
**Better equipped to engage and respond to men**			
	Improved insight into therapy with men	76 (19.4)	
	Better understanding of masculinity and gender (in general)	78 (19.9)	
	Better equipped to engage men	25 (6.4)	
	Male suicidality content	17 (4.3)	
	Male depression content (eg, MDRS^b^)	13 (3.3)	
**Content filled a gap**			
	Evidence-based and expert-delivered content	67 (17.1)	
	Relatable and relevant content	41 (10.5)	
	Practical skills	34 (8.7)	
	Validated existing learning	15 (3.8)	
**Engaging learning experience**			
	Worksheets and toolkit exercises	58 (14.8)	
	Engaging presentation and platform	58 (14.8)	
	Simple to work through	51 (13)	
	Prompted reflection and practice	27 (6.9)	
	Self-paced	17 (4.3)	
	Variety of learning formats	12 (3.1)	

^a^Percentage represents the percentage of participants who responded to the qualitative items (n=392) rather than the full RCT sample (N=587).

^b^MDRS: Male Depression Risk Scale.

#### Depictions of the Video Content in Men in Mind

The next category encompassed participants’ reflections on the apparent outcome of the training, in which many felt better equipped to engage and respond to men in therapy. A general sense of improved insight into the “male experience of therapy” by feeling better equipped to apply a “gender lens” was noted here. In particular, participants appreciated education regarding “strategies that are specifically [targeted] at men rather than generic counselling practices” and content that guided them on leveraging their gender to assist male clients. Practitioners (both men and women) also valued learning about the ways in which their own gender could be leveraged and positioned to assist male clients: “Deeper reflection on own gender – biases, gendered thinking, own experiences of masculinity growing up.”

The responses next suggested Men in Mind addressed a previously unmet need and filled a content gap. This included participants valuing the evidence-based nature of the content, alongside the provision of supplementary research to allow participants to expand on their learning: “links to the research articles—my male clients love it when I refer to or send them research.” Specifically, information on male depression screening, including how to use the Male Depression Risk Scale, was mentioned by many researchers [35]. Participants valued the “practical strategies” taught in Men in Mind, including “examples of what language to use and how to implement the theories in sessions,” which facilitated implementation: “I began to use this training in my practice immediately.”

Finally, the participants appreciated the engaging learning experiences provided throughout Men in Mind. The most highly favored aspects of the learning experience included the worksheet exercises (“the resource pack at the end was perfect”); alongside the presentation of the training content, which was described as “very aesthetic and engaging.” Furthermore, the training was described as simple to work through, with content that progressed logically (“logical flow from theory to assessment to engagement to intervention”) at an accessible pace that allowed completion “in [their] own time.”

#### Suggested Improvements to Men in Mind

Regarding improvements to Men in Mind, while the most commonly occurring code (93 participants) reflected praise of the program and a lack of areas for improvement (eg, “nothing—this was one of the best, most clearly informative and practically useful courses I have undertaken”), the 392 respondents to this item nonetheless suggested numerous areas for improvement (Table 4).

**Table 4:** The thematic analysis map of participants’ improvement responses.

Group		Responses, n (%)^a^
**General content improvements**		
	Greater quantity of content	62 (15.8)
	Greater depth of content	22 (5.6)
**Course format**		
	Unrealistic time allocation	49 (12.5)
	Note-taking improvements	34 (8.7)
	Improvements to learning aids (eg, worksheets)	63 (16.1)
	Technological issues (eg, saving progress and downloading files)	17 (4.3)
**Video improvements**		
	More (or longer, more diverse) video content	42 (10.7)
	Staged video or acting improvements	11 (2.8)
	Improvements in therapeutic techniques of practitioners in videos	6 (1.5)
	Subtitles on videos	6 (1.5)
	Unscripted (or more natural) videos to analyze	6 (1.5)
**Access and follow-up**		
	Unlimited or longer access to the training (after completion)	18 (4.6)
	Group learning or supervision; question and answer with facilitators	15 (3.8)
	Follow-up platform with emerging evidence or resources	13 (3.3)
	Ensure course is recognized by more organizations	8 (2)
**Content population focus**		
	More content on specific presentations (eg, alcohol or substance use, anxiety, PTSD^b^, ASD^c^, relationships, and IPV^d^)	15 (3.8)
	More content on gender and sexual diversity	9 (2.3)
	Specific modules for different age groups	8 (1.3)
	More cultural diversity	5 (1.3)
	More content on suicidality	6 (1.5)
	Content on male-specific presentations of resistance	2 (0.5)

^a^Percentage represents the percentage of participants who responded to the qualitative items (n=392) rather than the full RCT sample (N=587).

^b^PTSD: posttraumatic stress disorder.

^c^ASD: autism spectrum disorder.

^d^IPV: intimate partner violence.

The first subtheme regarding content improvements was related to suggested additional content regarding subgroups or presentations among men. Most commonly this not only referred to the need for more sexual and gender diversity (eg, “More focus on varied gender and sexual identities, e.g., transgender males, gay, etc.”) but also pertained to men of varying age groups (eg, “more content of and for adolescent males,” “additional age ranges”) and cultures (“Further emphasis on unique issues faced by men of colour...”). In addition, participants noted the potential for further content focused on a range of presenting issues among men, including suicidal thoughts or behaviors (eg, “I think it would be good to spend more time on suicide intervention, as Module 5 really could have been separated into two modules”) and men’s perpetration of abuse or interpersonal violence (eg, “didn’t really touch on any kind of abuse, violence, or offending behaviours which might be extremely difficult to respond to without prior training”).

Furthermore, common across responses was a suggestion to include additional content in specific forms (eg, “more visual and lecture-based content,” “more role plays and scripted techniques”) or provide greater depth regarding what some participants perceived to be a relatively simplistic depiction of men and masculinity: “The information felt a bit cliche to a particular stereotype of a very blokey Australian man and some of the tips and interventions then also felt they served kind of to reinforce this stereotype... I see quite a lot of men in my practice and some of the issues/themes only seemed relevant to a very small subset of these: very closed off, sporty, blokey, angry (for which they were extremely helpful by the way).”

Suggestions for improving the videos were also common across responses. This primarily concerned the suggestion for more videos, along with longer (eg, “Even more video examples, with different options or what is helpful to say or not say given the diverse clients presented”) or more varied scenes for participants to reflect on; “The videos/scenarios could represent more varied situations, not just a therapeutic setting. Such as support worker roles, social workers etc.” Some suggestions were also offered to improve the authenticity of the videos, “I’m just never a huge fan of staged session videos,” alongside accessibility improvements; “I think for accessibility purposes, the videos could include captions and also the feature to be sped up.”

Subsequently, improvements were made regarding the overall presentation and learning experience offered to the participants. This primarily concerned improvements regarding participants’ capacity to consolidate and retain their learning (eg, “Some forced recall might help me to integrate the knowledge,” “More quizzes and ways to check understanding and application of the content along the way”). Some participants noted the need for more time to engage with the training in greater depth (eg, “Higher allocation of time. It took me about twice as long as estimated”) with others suggesting improvements regarding the somewhat “clunky” in-built note-taking feature (“I think it would be great to leave space in the modules themselves for people to write responses”). A small number of participants mentioned issues surrounding the saving of their progress or the capacity to download provided readings or learning aids.

Finally, suggested improvements concerned the desire for opportunities to engage with the training material in more depth by discussing learnings and practicing skills with other practitioners (eg, “It would be awesome if there was an interactive component eg. Webinars and activities with other people, not just with recorded content and papers”). Participants suggested there would be value in additional “follow-up” or “refresher” training to aid in implementation of learning over time.

#### Confidence Following the Completion of Men in Mind

Finally, while it was common across responses for the 392 respondents to report universally improved confidence when working with men (67 participants; eg, “all men,” “most men actually”) participants reported improved confidence working with a variety of subgroups of men in therapy following the training. The most common category referred to improved confidence in working with men experiencing difficulty with emotional literacy and expression (eg, “Men who fit the stereotypical profile that limits emotional expression and authentic communication”), where participants often reflected a sense of improved confidence to unlock the internal world of “emotionally numb” men. Encouragingly, participants also reported improved confidence when “working with men in high risk/crisis situations,” particularly being more aware of signs of suicidality that “might not be expressed verbally by the client.” Improved confidence to respond to men’s reactivity, particularly anger, was also common (eg, “men who others see as having ‘anger issues’”), along with working with men with depression or anxiety (eg, “men who may not show the typical signs of depression”). Other specific subgroups of men were also mentioned (Table 5).

**Table 5:** The thematic analysis map of participants’ confidence responses.

Group			Responses, n (%)^a^
**Men with emotional difficulties or mental health issues**			
	Men experiencing difficulty with emotional communication or regulation	190 (48.5)	
	Men experiencing suicidality	107 (27.3)	
	Angry men, men with difficulty with aggression	43 (11)	
	Men with depression or anxiety	21 (5.4)	
**Other specific types of men**			
	Men with traditional (masculine) attitudes; gender role strain	32 (8.2)	
	Sexual minority men	31 (7.9)	
	Men from different cultural backgrounds	5 (1.3)	
	Incarcerated or violent men	5 (1.3)	
**Men of different ages**			
	Old or older men	29 (7.4)	
	Adolescent or young men	27 (6.9)	
	Middle-aged men or fathers	16 (4.1)	
	Men across the life span	2 (0.5)	
**Resistant or unmotivated men**			
	Detached, indifferent, ambivalent, or unmotivated men	17 (4.3)	
	Resistant, ambivalent, or reluctant men	15 (3.8)	
**Men impacted by significant life events**			
	Men struggling with role transitions (eg, job, relationship, and schooling)	12 (3.1)	
	Men experiencing trauma or abuse	3 (0.8)	
	Men experiencing chronic illness or physical losses	2 (0.5)	

^a^Percentage represents the percentage of participants who responded to the qualitative items (n=392) rather than the full RCT sample (N=587).

### Goal Assessment Data

**Figure 3:**
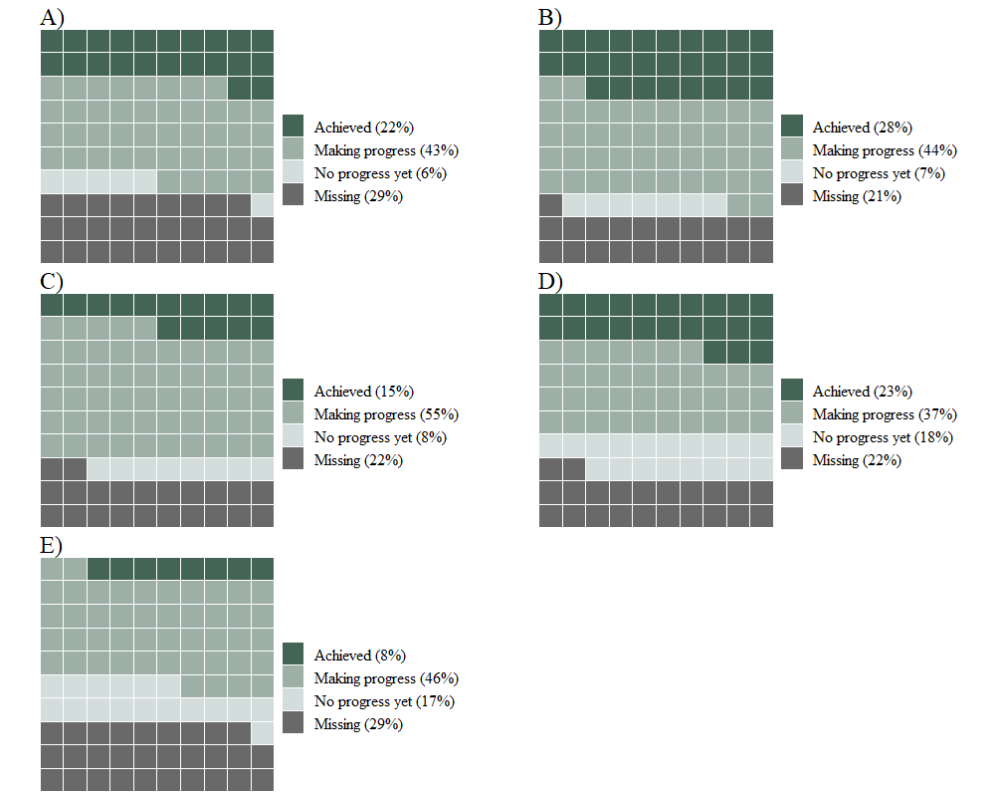
Achievement percentages for each of the 5 goal categories (N=603), as follows: A) Leverage masculinities (n=162), B) Improve enagement or retention (n=153), C) Men's emotions (n=60), D) Men's suicidality or depression (n=118), and E) Consolidate learning (n=110).

Participants in the Men in Mind group (n=204) provided up to 3 goals at T2, resulting in a total of 603 goal responses. Of these 603 individual goal responses, a further 453 were matched with self-reported goal achievement scores at follow-up. Figure 3 presents the 5 goal categories, frequencies, and the corresponding achievement ratings. The 5 goal categories related to goals aimed to *leverage masculinities in therapy* (exploring masculinity and the impacts of gender socialization), *improve engagement or retention* (specific male-oriented engagement strategies), *work better with men’s emotions* (strategies to assist men experiencing difficulty identifying or articulating their emotions), and *consolidate learning* (goals relating to retaining knowledge from the training). Multimedia Appendix 2 provides a more detailed description of these categories along with participant examples for each. Goals coded as belonging to the *improve engagement or retention* category were achieved at a higher rate than any of the other 4 categories. Similar, but slightly lower, achievement scores were noted for the *leverage masculinities in therapy* category. In contrast, goals coded under the *consolidate learning* category had the lowest achievement rates. Overall, 85.4% (387/453) of the goals with achievement ratings were rated as “achieved” or “making progress” at the 12-week follow-up.

## Discussion

### Principal Findings

This study investigated participants’ experiences of a web-based training program, Men in Mind*,* in depth, addressing previous gaps in intervention evaluation and collating data to assist in real-world scaling and implementation of this intervention. This study also aligns with international recognition of the value of mental health practitioner development in the psychology of men and masculinities to aid their practice and provides a concrete avenue to achieve this end [27]. Findings across the qualitative feedback items highlighted the current strengths of Men in Mind, particularly in terms of the value of video vignettes of skills in action, improved insight into the connections between traditional masculinity and the therapy environment, and the engaging learning experience provided. Suggested improvements largely reflect the need to expand the training material with more inclusivity, diversity, and equity-tailored content, along with necessary improvements to the provided learning aids and novel content areas to include in future iterations. Encouragingly, participants reported improved confidence in assisting men struggling with emotional communication and suicidal men. Complementing participants’ qualitative data, results from the quantitative feedback items showed consistency across domains of practitioner self-efficacy as having improved after Men in Mind, and importantly, that practitioners felt improvements in engaging and retaining men in their practice. Altogether, these findings indicate necessary improvements before the scaling of Men in Mind, while also informing viable components of practitioner e-learning interventions focused on men’s health service engagement.

### Qualitative Feedback

#### Successes of Men in Mind

According to participants, the 40 professionally produced role-play videos were the most enjoyable aspect of the program. This reflects the value of providing guided demonstrations, particularly when video content is segmented into clearly defined sections and key takeaways are highlighted via accompanying text [36]. The desire among therapist learners for observational learning has also been documented in prior research [37]. Coupled with the sourcing of topic areas for the videos directly from past evidence of practitioner challenges working with men [30], these factors likely contributed to the authenticity and resonance of the video content in Men in Mind. This was confirmed by broad feedback that Men in Mind was an engaging learning experience, reflecting the efforts that went into the program to incorporate best practice learning design methods [38-40]. For example, participants’ endorsement of the value of providing multiple learning tools (ie, videos, supplementary reading material) accords with prior research documenting the diversity in adult learning styles and the capacity of e-learning platforms to appropriately cater to this [41]. Finally, and perhaps most importantly, practitioners valued the improved insight into therapy with men provided by the Men in Mind intervention. This substantiates the identified gap in training for mental health practitioners, particularly regarding the sensitization of therapy for help-seeking men [6].

#### Suggested Improvements to Men in Mind

A key recommendation for improving Men in Mind was feedback to increase the amount and depth of the current content, integrating more targeted content for specific subgroups of men, as well as men who operate outside of traditional masculine norms. This is an important critique, as there is growing recognition of the essential role of intersectional approaches to men’s health promotion [42]. Diversity in the intersections of gender and other factors (eg, culture, socioeconomic status, age, and sexuality) in influencing men’s mental health outcomes (eg, suicide rates) was acknowledged throughout the training. However, specific recommendations for working with demographic subgroups of men were beyond the scope of this iteration of Men in Mind. Regarding intersectionality and men’s mental health, there is a lack of evidence-based approaches to educate practitioners in balancing the recognition of individual diversity while also targeting therapeutic engagement strategies based on clients’ social group membership without re-entrenching stereotypes. It is important to contextualize the unique dichotomy arising here, whereby men’s mental health is situated as a “specialized” area of practice yet relates to clinical considerations for a vast and diverse group (almost half of the global population). Future iterations of Men in Mind will aim to build on the current content to explore practice adaptation recommendations when working with particular subgroups of men, as informed by lived experience and expert consensus (eg, First Nations men and gender and sexual minority men). Moreover, while some practitioners suggested the need for more specific content, others referred to the length of the course as a barrier to completion, highlighting a point of tension. Providing a course that is sufficiently generalist to capture the interests and needs of many practitioners will ultimately fall short of practitioners looking for more specialist information. However, a significant part of the course length feedback was related to the time estimates provided for the program being referenced as inaccurate and potentially unrealistic. Therefore, more realistic time estimates at the start of the course could ease the tension between these 2 contradicting areas of feedback. Providing continued or unlimited access to the modules (after the 6-week period) was also pointed out by practitioners as a potential solution. Regardless, the feedback emphasized the need for learning materials in this area to expand from the baseline understanding provided by Men in Mind to specialist content focusing on male priority subgroups of interest.

#### Confidence to Work With Different Groups of Men

Participants most often reported improved confidence to assist men having trouble identifying or communicating their emotional experiences. This finding reinforces the common challenge experienced by practitioners in delivering therapy to male clients who ostensibly lack the emotional literacy deemed a requisite for engagement [30,43-46]. The emotional restraints socialized by traditional masculinities have been considered a critical factor in therapy being deemed the “antithesis of masculinity” [47]. That participants reported improved confidence in this domain reflects the value of emphasizing the onus on practitioners to appropriately sensitize their therapy for men, taking on available recommendations for effective strategies to improve men’s emotional literacy [48]. Participants also commonly reported improved confidence to respond to men’s anger. These findings potentially reflect Men in Mind’s predominant focus on traditionally masculine identifying men, for whom difficulty with emotional expressions of distress via anger can be common [49]. While this was intentional given evidence substantiating traditional masculine norms as barriers to mental health service engagement [50-52], it is important for future iterations of Men in Mind to further emphasize inclusivity and plurality in masculinities to assign a diversity of agency in response to wide-ranging structures and masculine norms [53,54]. In addition, considering the evidence of high rates of mental health service contact before suicide in men [55], participants also reported improved confidence in assisting men experiencing suicidality. This is particularly reassuring given previous evidence that many practitioners feel less competent to work with suicidal men (relative to women [56]). As many practitioners grapple with a small window of opportunity to work with suicidal men, compromised due to delayed presentation to health services [57], improving practitioner confidence to capitalize on men’s engagement is essential.

#### Participant Goals and Achievement Scores

Likely reflecting the novelty of applying a “gender lens” to their therapy engagement strategies, the most commonly observed category of participants’ goals following completing Men in Mind was to leverage masculinity to engage their male clients. This is noteworthy given the extent to which traditional masculinity has often been pathologized in existing modes of therapeutic engagement as a unitary construct categorically incompatible with psychotherapy [18]. The appetite among participants to implement their learning in this domain, evident in their goals to leverage masculinity, is encouraging in light of long-standing recommendations for mental health systems and services to adapt to men rather than vice versa [58]. Participants also consistently reported goals to improve their engagement strategies, likely reflecting the vast range of strategies offered to engage men, derived from prior research [31]. In addition, goals to consolidate learning likely reflected the broad array of supplementary reading provided in the intervention, suggesting an appetite among participants to broaden their familiarity with men’s mental health literature.

In terms of goal achievement ratings overall, it was most common for participants to report achieving goals related to improving their engagement and retention strategies. Perhaps these goals were more readily achieved because of the highly practical nature of the training material: participants are presented with a logical framework of engagement strategies to facilitate seamless implementation. Similarly, the practical strategies offered to engage male clients in discussions around the potential intersection between traditional masculine norm adherence and their mental health likely facilitated participants’ achievement of their goals around leveraging masculinity. Notably, the goals made regarding consolidating learnings from the course had the lowest rates of achievement. While the other 4 goal categories centered around improvements for working with men in therapy, the learning consolidation category focused on improving and retaining knowledge from the course. This could signify that it was challenging for participants to retain theory-based knowledge around the course, a factor inhibiting the integration of these frames into practice. This aligns with the qualitative feedback regarding the best elements of Men in Mind where practitioners commonly highlighted the role-plays and practical demonstration of skills as the most compelling part of the program.

### Implications

Previous findings in the field of e-learning suggest that centering on learner experience in the development of e-learning interventions can be a critical component of engagement [59]. In the case of practitioner training for engaging and responding effectively to help-seeking men, the uptake of training material was critical in light of the lack of available avenues to address established disconnects between practitioner training and men’s mental health service engagement. The positive user experience feedback results found in this study, when considered alongside the high level of engagement and improvements in practitioners’ clinical competencies [22], reinforce the critical value of learning and user experience design input alongside extensive user testing with the practitioner group when developing e-learning interventions. This is particularly relevant when considering the rollout and implementation of e-learning programs on a broader scale, with the efficacy of Men in Mind supporting claims that having this design input and user testing can be essential to ensuring user desirability and long-term integration into clinical practice [59]. The findings also suggest the value of adopting learnings from the Men in Mind model to develop more widespread practitioner training in other areas of neglected focus for boys and men’s mental health promotion, particularly when masculine norm adherence is thought to play a role in the exacerbation of poor outcomes. Examples include engaging boys and men in therapy to assist recovery from childhood sexual abuse, where often vast delays in help-seeking can arguably increase the necessity for practitioners to effectively capitalize on early windows to establish therapeutic alliance [60].

Three key areas of interest could be improved in future iterations of the program. First, we aimed to improve the expectation setting at the start of the program, specifically with regard to more realistic time estimates for completion, and how the program should be contextualized (ie, as a foundational course for working with men generally, before pursuing further information regarding the range of subgroups within men). This is directly in response to feedback regarding improving content depth and course length, where most participants were happy to complete a longer course but were unaware of what they perceived as an unrealistic time estimate. Second, we aim to improve access to the program by improving the depth and variety of the take-home materials practitioners who are given after completion of the program. Accessibility is a critical component of web-based learning, and extended access to course content would allow for further repeated use of the content, reinforcement of information and practice, and the ongoing implementation support necessary to ensure implementation fidelity [61]. Finally, to assist participants in their shared learning and implementation of the Men in Mind content, there is likely value in the establishment of a community of practice to facilitate group supervision and ultimately contribute to a gender-sensitive practitioner workforce more broadly.

### Limitations and Future Directions

The limitations of this study include its reliance on participants’ self-reports and the use of novel questionnaire items. This limits the extent to which we can infer tangible changes in practitioner behavior; however, our approach was intentional, given the qualitative design and aims to obtain an in-depth understanding of participants’ experiences with Men in Mind. Therefore, the reliance on self-report data could also be considered a strength of the study, in that practitioner self-report is an effective way to tap into the depth of user experiences. There was also a significant difference in age and experience for participants who provided feedback and goal assessment data compared with our original RCT sample. This may imply that the results of this study are not representative of younger, less-experienced practitioners, which is a limitation considering that younger and less-experienced practitioners might be most amenable to behavior change via e-learning interventions. An obvious gap also concerns the extent to which improvements to practitioner confidence and their achievement of learning goals translates to improvements in outcomes among male clients (eg, improved engagement in care). Our demonstration of the efficacy of Men in Mind via RCT [22], coupled with qualitative evidence from this study of participants’ favorable experiences with the intervention, solidifies the testing of client outcomes as a viable next step. Indeed, consultation with male clients [9] has confirmed that health services and practitioners not responding appropriately to the needs of male clients is a critical problem in this field. Ensuring that Men in Mind is successfully addressing this problem, from the perspective of the male clients themselves, is essential.

### Conclusions

In this study, we have reported in-depth experiences with the Men in Mind training intervention, including the best elements and those in need of improvement. Participants reported improved confidence in engaging a range of help-seeking men and encouragingly reported clear goals for implementing their learning into practice. Our findings substantiate the need for effective knowledge translation efforts that bridge the gap between evidence and practice. When focusing on mental health service delivery, such approaches do due justice to the complex and often systemic issues that can stymie mental health service engagement and outcomes.

## Appendix

Multimedia Appendix 1 Comparison of respondents and nonrespondents on baseline demographics.

Multimedia Appendix 2 Examples for each of the 5 goal categories reported by participants in the Men in Mind group.

## Abbreviations

RCT: randomized controlled trial

## Data Availability

The data sets generated during this study are not publicly available due to ethical restrictions, but data are available from the corresponding author upon reasonable request and with ethics approval for secondary analyses.
