# Supplementary material for: Supporting Clinical Competencies in Men’s Mental Health Using the Men in Mind Practitioner Training Program: User Experience Study
Source: JMIR Med Educ. 2023 Nov 7;9:e48804. doi: 10.2196/48804 (PMC10664012; doi:10.2196/48804)
Supplement: Multimedia Appendix 1 [file mededu_v9i1e48804_app1.docx]

**Multimedia Appendix 1**

**Table 5.** Respondents vs. non-respondents comparison based on baseline demographics

|  | Comparison with baseline demographics (*N*=587) | | | |
| --- | --- | --- | --- | --- |
|  | Quantitative items | Qualitative items | Goal categories | Goal achievement |
|  | *n*=395 | *n*=392 | *n*=204 | *n*=152 |
| Age (*t*, *P*) | *t*=3.38, *P=*.001 | *t*=3.31, *P=*.025 | *t*=2.25, *P=*.025 | *t*=2.17, *P=*.031 |
| Gender^a^ (*χ*^2^, *P*) | *χ*^2^=3.13, *P=*.209 | *χ*^2^=2.33, *P=*.312 | *χ*^2^=2.62, *P=*.270 | *χ*^2^=1.15, *P=*.562 |
| profession^b^ (*χ*^2^, *P*) | *χ*^2^=12.35, *P=*.090 | *χ*^2^=11.02, *P=*.138 | *χ*^2^=5.28, *P=*.626 | *χ*^2^=4.70, *P=*.696 |
| Years of experience^c^ (*χ*^2^, *P*) | *χ*^2^=8.02, *P=*.046 | *χ*^2^=9.40, *P=*.024 | *χ*^2^=6.56, *P=*.088 | *χ*^2^=1.62, *P=*.654 |
| Employment load^d^ (*χ*^2^, *P*) | *χ*^2^=3.72, *P=*.294 | *χ*^2^=3.27, *P=*.353 | *χ*^2^=1.49, *P=*.685 | *χ*^2^=1.28, *P=*.735 |
| Qualifications^e^ (*χ*^2^, *P*) | *χ*^2^=2.96, *P=*.706 | *χ*^2^=2.29, *P=*.808 | *χ*^2^=3.24, *P=*.663 | *χ*^2^=3.20, *P=*.670 |
| Workplace^f^ (*χ*^2^, *P*) | *χ*^2^=8.43, *P=*.393 | *χ*^2^=7.66, *P=*.467 | *χ*^2^=9.27, *P=*.320 | *χ*^2^=10.20, *P=*.251 |
| Region^g^ (*χ*^2^, *P*) | *χ*^2^=1.99, *P=*.370 | *χ*^2^=1.32, *P=*.516 | *χ*^2^=1.63, *P=*.444 | *χ*^2^=1.90, *P=*.386 |

^a^ Gender = male, female, self-identified gender.

^b^ Profession = provisional psychologist, general psychologist, clinical psychologist, counsellor/psychotherapist, occupational therapist, social worker, nurse practitioner, family therapist/practitioner.

^c^ Years of experience = 0-2 years, 3-5 years, 6-10 years, 11+ years .

^d^ Employment load = full time, part time, casual/contractor, other.

^e^ Qualifications = Certificate 3, Certificate 4, Undergraduate degree, Undergraduate degree (Hons.), Master’s degree, Doctoral degree/PhD .

^f^ Workplace= Public or community health service, private practice, hospital, corporate organisation, educational service (e.g., school, university, TAFE), not-for-profit organisation, prison or correctional facility, veterans’ mental health service, government or government organisation.

^g^ Region = metropolitan, regional, rural/remote.
