# Supplementary material for: Supporting Clinical Competencies in Men’s Mental Health Using the Men in Mind Practitioner Training Program: User Experience Study
Source: JMIR Med Educ. 2023 Nov 7;9:e48804. doi: 10.2196/48804 (PMC10664012; doi:10.2196/48804)
Supplement: Multimedia Appendix 2 [file mededu_v9i1e48804_app2.docx]

**Multimedia Appendix 2**

**Table 6.** Examples for each of the five goal categories, *Men in Mind* group participants

| Category and description | | Examples (s) |
| --- | --- | --- |
| Leverage masculinities in therapy | |  |
|  | Goals that broadly reflected goals pertaining to helping men explore their sense of masculinity in session and how this might be impacting their lives. | “*to use a gender-focused lens whilst working with men*”  “*To be able to have a conversation about the impact of masculinity on a man’s experience of distress*” |
| Improve engagement/retention | |  |
|  | Goals that encompassed planned implementation of specific male-oriented engagement strategies taught in *Men in Mind.* | “*Make it clear what can be expected - e.g. in the first session discussing how long we might be doing therapy for*”  “*To really highlight the collaborative nature of therapy for the men with whom I work in order to increase their sense of control.*” |
| Work better with men’s emotions | |  |
|  | Goals that reflected implementation of strategies taught in Men in Mind to assist men experiencing difficulty identifying or articulating their emotional experiences. | “*Encourage identification and articulation of emotions*”  “*exploring anger directly first .. not rushing to what may lie underneath*” |
| Work better with men’s depression and suicidality | |  |
|  | Goals that related to improving their identification and responses to men’s depression and suicide risk in session. | “*Use the male-specific depression questionnaire when assessing for depression*”  “*Working with men who are feeling suicidal; looking into the warning signs such as Anger/Agression and Risk Taking and then responding to them with strengths based open questions to assess the risk and explore the underlying issues*” |
| Consolidate learning | |  |
|  | Goals that rarely referenced a specific section of *Men in Mind*, but often included reference to the various learning aids, and surrounding literature provided, alongside general goals to revisit the training. | “*Re-read through journal articles and resources given during the training*”  “*Review the training material*” |
